# Exploring the Functional Potential of Date (*Phoenix dactylifera*) Seed Bioactives in Modulating Gut Microbiota in Diabetic Rats

**DOI:** 10.1002/fsn3.71841

**Published:** 2026-05-03

**Authors:** Nouf Abdullah Alharbi, Saleh A. Alsanie, Asmahan A. Ali, Aeshah Alhosain, Tarfa Albrahim, Thamer Aljutaily, Huda Aljumayi, Isam A. Mohamed Ahmed, Belal M. Mohammed, Nazeha A. Khalil

**Affiliations:** ^1^ Department of Basic Health Sciences, College of Applied Medical Sciences Qassim University Buraydah Saudi Arabia; ^2^ Department of Food Science and Human Nutrition, College of Agriculture and Food Qassim University Buraydah Saudi Arabia; ^3^ Department of Health Sciences, Clinical Nutrition, College of Health and Rehabilitation Sciences Princess Nourah Bint Abdulrahman University Riyadh Saudi Arabia; ^4^ Department of Food Science and Nutrition, College of Sciences Taif University Taif Saudi Arabia; ^5^ Department of Food Science and Nutrition, College of Food and Agricultural Sciences King Saud University Riyadh Saudi Arabia; ^6^ Department of Food Science and Technology, Faculty of Agriculture and Food Sciences Ibb University Ibb Yemen; ^7^ Nutrition and Food Sciences Department, Faculty of Home Economics Menoufia University Shibin El Kom Egypt

**Keywords:** date seed by‐products, lipid profile, triglyceride *Bifidobacterium* and *Lactobacillus*

## Abstract

Date fruits (*Phoenix dactilyfera* L.) are widely produced with huge wasted by‐products (seeds/pits and fibers) in remarkable nutritional and economic potential value. This study aims to examine the impact of the functional properties of date seeds on health status and gut microbiota (GM) modulation in diabetic rats. Collected samples were dried and used in powdered form (DSP) for chemical compositions. Diabetic rats were then fed with this (5%, 10%, and 15%) versus control healthy group. Blood samples were used for glucose (BG) with lipid profiles (cholesterol; CHO), triglycerides (TG), high‐density lipoprotein (HDL) and low‐density lipoprotein (LDL). Additionally, kidney (Urea, Criatinine) and histological function and GM were measured. Data indicated that DSP contain crude fiber, total sugar, and crude fat about 77%, 6%, and 6% respectively. Additionally, potassium was the highest while phosphorus shows the lowest (31.53 ± 1.19 vs. 2.48 ± 0.31 g/100 g DSP). Iron, calcium, sodium, and magnesium were 14.29 ± 0.59, 12.53 ± 0.49, 9.23 ± 0.22, and 7.60 ± 0.42 g/100 g DSP respectively. The 10% and 15% DSP supplementations affected the GM; *Clostridium histolyticum* reductions versus *Bifidobacteria, Lactobacillus* enhancement. Also, kidney functions and lipid profile improved especially at 15% supplementations (CHO and TG decrease vs. HDL‐c increase). Valorization of date seed by‐products indicates their potential health effects on lipids and kidney functions among diabetic models influenced by their antioxidant, mineral and fiber levels in association to GM composition. However, further studies are needed on humans with sustainable utilization manner of food waste.

## Graphical abstract

Date fruits (*Phoenix dactilyfera* L.) are widely produced with huge wasted by‐products (seeds/pits and fibers) in remarkable nutritional and economic potential value. This study aims to examine the impact of the functional properties of date seeds on health status and gut microbiota (GM) modulation in diabetic rats. Valorization of date seed by‐products indicates their potential health effects on lipids and kidney functions among diabetic models influenced by their antioxidant, mineral, and fiber levels in association to GM composition.

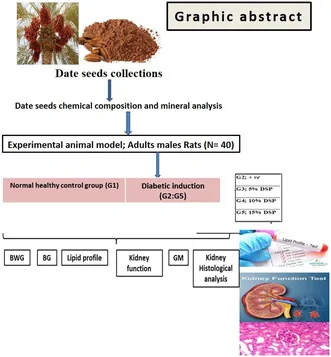

## 1 Introduction

Type 2 diabetes mellitus (T2DM) is a common metabolic disorder globally characterized by hyperglycemia (high blood glucose levels). It is caused by insulin resistance and/or insulin secretion reduction (Ibrahim et al. 2023). Again T2DM has become a global health problem due to the rise in morbidity and mortality. Intervention measures focus mainly on glucose control. However; prevention and treatment of related complications cannot be cured (Zhou et al. 2022). Growing evidence suggests that gut microbiota (GM) dysbiosis is defined as the gut microbial imbalance community associated with many health disorders; like inflammatory bowel disease, obesity and metabolic diseases T2DM (Acevedo‐Román et al. 2024). Indeed, previous studies have proven that T2DM is often associated with fecal microbiota dysbiosis or dys‐regulation elevating the intestinal permeability through energy and fatty acid metabolism in addition to glucose homeostasis and insulin sensitivity (Mendis et al. 2025). Also, published review presented that GM metabolites are involved in pathogenesis and characterization in T2DM population as well as diabetic animal models (Zhou et al. 2022). Therefore, it is important for the human health to modulate the GM for glycemic control improvement and insulin sensitivity enhancement. It also could lower the fasting blood glucose levels that in total could reduce gut permeability, dampen systemic inflammation, and improve intestinal barrier integrity (Acevedo‐Román et al. 2024). However, it could be modified for metabolites secretion in a special engagement called host gut‐microbe crosstalk by derived metabolites such as short‐chain fatty acids (SCFAs; formed during the microbial fermentation of fiber) especially butyrate, acetate, and propionate (Khalil et al. 2013; Bui et al. 2025). Bile acids, lipopolysaccharide tryptophan, glycemic control, insulin sensitivity enhancement and fasting blood glucose reduction‐ especially by dietary prebiotics supplementations and/or interventions are needed (Gibson et al. 2017). The term prebiotics is well known for the non‐digestible foods promoting the growth and activities of beneficial gut microbiota. Probiotics have been known as “live microorganisms” and both can be used together as a term of synabiotic to beneficially affect the host (Ooi and Liong 2010).

Dietary fibers are known as an indigestible carbohydrate; part in plant‐based foods. Both support different aspects of human health such as bowel regularity, glycemic control, and cholesterol management. Also, they can help restore the balance in the GM then reduce the oxidative stress possessing antioxidant activity and contributing to overall gut health (Guo et al. 2024). In total, it is comprised of substrates conferring different health benefits but may be utilized by host microorganisms selectively. For example, high dietary fiber with more fruits and vegetables improves the intestinal microflora in addition to sugar reduction levels (Bacha et al. 2024). Also, dietary fibers play critical roles in glycemic regulation with GM association by glucose absorption reduction (maintaining glucose homeostasis) in the small intestine that enhances insulin sensitivity. Responses contribute to both the prevention and management of T2DM significantly (Acevedo‐Román et al. 2024). Therefore, the dietary fibers have prebiotic effects by binding to bile acids in the gut leading to increased bile acid excretion and reducing circulating levels subsequently of low‐density lipoprotein cholesterol (LDL‐c) and triglycerides decreased levels. This causes lipid profile improvements (Ooi and Liong 2010).

Date fruit (Phoenix dactilyfera L) is an important rich dietary fiber source (e.g., pectin, β‐glucan, and arabinoxylan). It contains high levels of sugars, polysaccharides, and bioactive compounds like carotenoids, flavonoids, phenolics, and anthocyanins; exhibiting prebiotic properties and supporting beneficial gut microbiota in addition to different minerals and vitamins that have antioxidant and anti‐inflammatory activities. All possess preventive and therapeutic influences against diseases in prebiotic potential effects (Alhomsi and Kılıç Bayraktar 2025; Alalwan et al. 2020; Alsarayrah et al. 2023). The worldwide date production, according to Food and Agriculture Organization Statistics, has escalated over the past decade (FAO 2023); from 7.53 to 9.6 million metric tons. The date seed waste gives 852 thousand tons approximately every year to post‐harvest loss (Jayasree et al. 2024). Seeds can introduce harmful substances into the environment. Still, they possess huge functional properties/activities such as anti‐inflammatory, antidiabetic, antibacterial, antioxidant activities and much more health‐promoting qualities (Begum et al. 2024; Jayasree et al. 2024). The supplemented levels Jubayer et al. (2020) were 600 mg/day for 90 days in a clinical trial on patients with hyperlipidaemia showing total cholesterol, triglycerides, LDL‐c and HDL‐c improved levels. Also, another study revealed that 2.5 g of date seed consumptions for 14 days decreased the expression of interleukin‐1β, transforming growth factor β, cyclo‐oxygenase‐1 and cyclo‐oxygenase‐2 in middle‐aged women (Saryono et al. 2020). The gut microbiota compositions may be affected as their polysaccharides resist digestion typically until reaching the colon. Then, they can support the growth of different beneficial bacteria; especially the probiotic species in order to contribute for SCFA production (Han et al. 2022). Such fermentation process can cause bowel health improvement and disease prevention.

Date seeds are wasted by‐products reported previously in antioxidant defense as positive results in addition for being used as coffee substitutes (Moslemi et al. 2022). However, to date, nothing has been reported anywhere of the evidence supporting date seeds' prebiotic activities in association with gut microbiota compositions and/or activities in diabetic models. Therefore, the current study has aimed to investigate the effects of date seeds in association with GM compositions in diabetic rats; mainly their impact on *Clostridium histolyticum, Bifidobacteria*, and *Lactobacillus* growth levels at different time points. It pointed to measure the seeds' effects on blood glucose, lipid profile, and kidney functions.

## 2 Material and Methods

### 2.1 Date Seeds Collections and Preparations

*Sukarry* dates were acquired from the local market, Giza, Egypt. Then the seeds have been separated manually, cleaned, and dried in sunlight for 1 day before being roasted. Medium temperature was used, then the seeds were crushed and powdered in a blender before being stored at 4°C. The date seed powders (DSP) have been used in feeding diabetic rats at different levels (5%, 10%, and 15% of the rats' diet as follows: with some of our different published papers, Khalil et al. 2023; Aljutaily et al. 2022). The used doses in the current study are calculated from the rats' food daily intake that is about 15–20 g diet in total/day. The 5% of 20 g should be about 1 g/day, while the previous recent researchers used 2.5 g date seeds/day for dietary intervention in human study (Saryono et al. 2020). Thus, the used doses were maximized to 15% as safe in the current study.

### 2.2 Date Seeds Chemical Composition and Mineral Analysis

Powdered date seeds have been analyzed for their chemical compositions including ash, moisture content, crude protein & fiber, and lipid levels as described by the methods in Association of Official Analytical Chemists (AOAC 2000). The measured components included the moisture content and carbohydrates (Nitrogen free extract) by an electric air draught oven (VEB MLW Medizinische, Gerete, Berlin, Germany) and by the difference respectively as described by our published data. Regarding the mineral measured contents, the following elements have been measured (potassium, K; sodium, Na; calcium, Ca; ferrous, Fe; magnesium, Mg; and zinc, Zn) by an Atomic Absorption Flame Emission Spectrophotometer (Perkin‐Elmer Model AA‐6200 from Shimadzu 7000, Japan) as reported by Association of Official Analytical Chemists (AOAC 2000).

### 2.3 Experimental Animal Model

Forty albino male rats (*n* = 40 weighted 115–123 g) were acquired from Experimental Animal Care, National training center, Cairo, Egypt. Total rats were fed normal control diet for 1 week as adaptation period. The normal control diet was formulated for rodent growth according to the American Institute of Nutrition recommendation and as followed and described by our previous studies (Aljumayi et al. 2024). The current study has been ethically approved by the Scientific Research Ethics Committee (Animal Care and Use; approval number 21‐SRE‐02‐2023), Department of Nutrition and Food Science, Faculty of Home Economics, Menoufia University, Shebin El‐Kom, Egypt. All rats were divided into two main groups in double‐blinded simple and random procedures (critical standard used to eliminate selection and detection bias ensuring that experimental results are reproducible). The first was normal healthy group (*n* = 8). They were fed control normal diet and used as control negative healthy group (G1). The second group was used for diabetic induction. Rats were injected intra‐peritoneally (IP) with 30 mg/kg of streptozotocin (single dose) as described recently by Ibrahim et al. (2023). The diabetic rats group has been subdivided into four sup‐diabetic groups (G2:G5; *n* = 8 rats each) as described in Table S1.

All rats had free water access and diet up to 28 days with different dietary supplementations as described in Table S1. Also, all the rats were weighed before and after establishing the study then the body weight gained (BWG) was calculated in addition to the blood sample collections for further analysis. Again, the fecal samples were collected for the gut microbiota evaluations while organ samples for kidneys were collected and used for histological structure evaluations.

### 2.4 Body Weight (BWG %) Measurements

At the beginning of the experiment, all rats' body weights were recorded and again at the end. Then, the groups' body weight gain (BWG %) was measured (comparing to the control diabetic positive group according to one of our previously published studies; Khalil et al. 2023) using the following formula:

$$\mathrm{BWG}\;\left(\%\right)\frac{\text{Final weight}–\text{initial weight}}{\text{Initial weight}}\times 100$$

### 2.5 Blood Glucose Evaluations

Serum blood glucose (BG) levels were carried out as described according to the method of Khalil et al. (2023) within the following analytical procedures that were adjustment to the method followed by Khalil et al. (2021) and Aljutaily et al. (2022).

### 2.6 Lipid Profile

Blood lipid profile and kidney functions were determined as described by Khalil et al. (2023) for serum total cholesterol (CHO), serum triglyceride (TG) and high‐density lipoprotein cholesterol (HDL‐c). Very low‐density lipoprotein cholesterol (vLDL‐c) was calculated using the following formula: vLDL‐c (mg/dL) = TG/5 in mg/dL. The low‐density lipoprotein cholesterol (LDL‐c) was calculated as LDL = CHO – (HDL‐c + vLDL‐c). Also, serum urea with creatinine determinate for kidney functions were measured as described previously by our published data (Nazeha et al. 2023) with few adjustments to the method followed by Khalil et al. (2024); Aljumayi et al. (2024).

### 2.7 Gut Microbiota Composition Measurements

The collected fecal samples were collected at 0 and 24 h at the final stage of 28 days. They were used for evaluating the colonic microbiota compositions. The measured composition species were total number, *Bifidobacteria, Lactobacillus*, and *Clostridium* as described before by our published data using flournace *in suit* hypridization (FISH) method (Khalil et al. 2013, 2021). The FISH technique used in the current study is a powerful tool for visualizing and counting specific microbial populations. However, it has several limitations leading to inaccurate quantification, like the probe hybridization bias. Additionally, it requires fixation and permeabilization of cells, and that can alter cellular morphology and potentially affect results. Furthermore, this technique is typically limited to detecting specific known sequences, making it less suitable for discovering novel genetic abnormalities. Thus, these limitations should be considered when interpreting the study's findings; however, it is the only available methodology at our convenience.

### 2.8 Histology Structure Examinations

The collected organ samples for kidneys have been used for histological structure evaluations. All kidneys fixed in 10% neutral formalin got buffered after being dehydrated in alcohol and clear in xylol then processed routinely and embedded in wax paraffin. All prepared sectioned paraffin blocks of 4–5 μm thickness were described within previous published data got stained with Hematoxylin and Eosin (Khalil et al. 2021).

### 2.9 Statistical Analysis

All the collected measured data have been presented as mean ± standard deviation (SD) in addition to using one‐way ANOVA (analysis of variance) for the differences between all group samples while post hoc test of Duncan's multiple range were used for the differences between pairs of means subsequently. All the used analysis carried out using SPSS software, version 21.0 (SPSS Inc., Chicago, IL, USA) and considered significant statistically at *p* ≤ 0.05.

## 3 Results

### 3.1 Chemical Composition and Measured Minerals of Date Seeds Powder

The measured chemical composition of date seeds powder (DSP) was presented in Table 1 for moisture, ash, crude protein, lipids, crude fiber, and nitrogen free extract while measured minerals were illustrated in the same table for potassium, calcium, sodium, magnesium, iron, and zinc (Table 1; *n* = 3 for each collected sample).

**TABLE 1:** The evaluated levels of date seeds chemical composition and minerals.

Chemical composition (%)		Mean of minerals (g/100 g powder)	
Moisture	3.29 ± 0.07	Potassium (K)	31.53 ± 1.19
Ash	3.01 ± 0.08	Calcium (Ca)	12.53 ± 0.49
Crude fat	5.99 ± 0.01	Sodium (Na)	9.23 ± 0.22
Crude protein	5.09 ± 0.01	Magnesium (Mg)	7.60 ± 0.42
Crude fiber	76.77 ± 0.06	Iron (Fe)	14.29 ± 0.59
Total sugars	5.86 ± 0.03	Phosphorus (P)	2.48 ± 0.31

*Note:* Date presented as mean ± SD, *n* = 3.

The data collected in Table 1 estimated that the used powdered date seeds (DSP) contain about 77% crude fiber that is the biggest levels between all the chemical compositions. Also, the seeds composition has total sugar percentages at about 6% and that are at the same levels roughly of crude fat (contains 6% approximately). Also, both fats and sugars show similar amounts. Additionally, the crude protein level is more than 5% and all of them are at high levels in contrast to moisture and ash levels (3.29% and 3.01% respectively; Table 1). Additionally, similar levels in Table 1 again demonstrated mineral levels of DSP. This shows the highest presented mineral with potassium (31.53 ± 1.19 g/100 g PDS) while the lowest were seen with phosphorus (P; 2.48 + 0.31 g/100 g DSP). Moreover, iron (fe), calcium (ca), sodium (Na) and magnesium (Mg) were at high presented levels at 14.29 ± 0.59, 12.53 ± 0.49, 9.23 ± 0.22 and 7.60 ± 0.42 g/100 g DSP (Table 1).

### 3.2 Body Weight and Blood Glucose Evaluations

Table 2 is illustrating the body weight gain of all used animal models at the beginning and the end of running the experiment. It shows that the first healthy rats' group (−ve; G1) fed on a basil diet has a mean of 120 g and that was increased to about 135 g by the end of the study. Therefore, such rats group has increased by about 15 g with body weight gain in about 12% (Table 2).

**TABLE 2:** The body weight gain of used animal models.

Rat groups	Initial body weight (g)	Final body weight (g)	Body weight gain (BWG; g)	% BWG (g)
G1; −ve	120.00 ± 1.79^a^	134.33 ± 3.72^b^	14.33	11.94
G2; +ve	120.33 ± 0.52^a^	113.00 ± 1.55^a^	−7.33	−6.09
G3; 5% DSP	123.33 ± 1.37^a^	134.00 ± 0.89^b^	10.67	8.65
G4; 10% DSP	122.67 ± 2.25^a^	138.33 ± 1.37^b^	15.67	12.77
G5; 15% DSP	122.33 ± 1.37^a^	142.67 ± 1.86^c^	20.33	16.62

*Note:* Presented value levels are mean ± SD; *n* = 8 each group. Different superscript letters under the same column bearing are significantly different (*p* ≤ 0.05).

* Note that DSP is the powdered date seeds.

Additionally, the other four groups induced with diabetes were nearly in the same body weight at the start point; about 120 g with no significant differences. However, their body weight changed at the end of the experiment with each treated group. The lowest was the positive diabetic rat group on a basil diet and that was decreased at the end of the study by about −8 g (about 7% BWG reductions). Also, treated groups by date seeds (DSP) in different levels (5%, 10%, and 15% additions) presented increased body weight levels significantly (*p* ≤ 0.05). The biggest group of rats was the rats fed 10% DSP supplementations while the lowest was the rats group fed 5% DSP (about 20 g vs. 11 g respectively) of their BWG significantly (*p* ≤ 0.05). However, all DSP supplementations helped body weight recovery in comparison to the positive diabetic rat group (G2; +ve).

### 3.3 Blood Glucose Evaluations

The following table illustrates the blood glucose levels in mg/dL collected from serums of used experimental rats at different time points; especially at the beginning or initial times and at the end of the experimental time point. Additionally, it shows the differences between both points and the differences from the control positive rats (G2; +ve). It has been revealed that the initial glucose levels were low in the healthy control normal group in contrast to the diabetic rats (G1 vs. all other groups). Their levels are 108 versus about 112 mg/dL respectively. Also, the same Table 3 reflects the effects of different DSP doses (5%, 10%, and 15%) on collected serum glucose levels significantly (*p* ≤ 0.05). They varied at the final time points while it was nearly the same effect on healthy group rats (after 28 days).

**TABLE 3:** The serum blood glucose levels of used animal models.

Rat groups	Initial blood glucose (mg/dL)	Final blood glucose (mg/dL)	Differences between initial and final; mg/dL	Differences from control (+ve); mg/dL
G1; −ve	108.00 ± 2.18^a^	111.33 ± 2.08^a^	3.33	−99.50
G2; +ve	210.33 ± 2.02^b^	210.83 ± 3.75^e^	0.50	0.00
G3; 5% DSP	214.00 ± 1.50^b^	195.33 ± 5.20^d^	−18.67	−15.50
G4; 10% DSP	213.33 ± 2.36^b^	160.50 ± 5.07^c^	−52.83	−50.33
G5; 15% DSP	212.00 ± 2.29^b^	121.83 ± 2.25^b^	−90.17	−89.00

*Note:* Presented value levels are mean ± SD; *n* = 8 each group. Different superscript letters under the same column bearing are significantly different (*p* ≤ 0.05).

* Note that DSP is the powdered date seeds.

Additionally, Table 3 has revealed that the control positive diabetic rats are nearly the same glucose levels (about 210 mg/dL) with no special effects while it was decreased after all the DSP supplementation significantly (*p* ≤ 0.05). The lowest effective DSP dose was seen with glucose levels of rats group fed 5% DSP (about 19 mg/dL; G3) while the biggest significant decrease (*p* ≤ 0.05) levels were at about 90 mg/dL with the highest reduced glucose amounts with rats fed 15% DSP even when compared with the positive control levels in significantly different (*p* ≤ 0.05; about −90 mg/dL) as shown in Table 3.

### 3.4 Blood Lipid Profile

The collected serum samples have been used for measuring the lipid profile levels (CHO, TG, HDL‐c, LDL‐c and vLDL‐c) of used animal models fed DSP and the data have been presented in Table 4.

**TABLE 4:** Lipid profile levels affected by DSP supplementation in used animal models.

Rat groups	Lipid profile (mg/dL)				
	Total cholesterol (CHO)	Triglycerides (TG)	High‐density lipoprotein (HDL)	Low‐density lipoprotein (LDL)	Very Low‐density lipoprotein (vLDL)
G1; −ve	68.37 ± 0.67^a^	29.21 ± 0.76^a^	58.36 ± 0.95^d^	4.16 ± 0.61^a^	5.84 ± 0.15^a^
G2; +ve	103.13 ± 1.63^e^	118.81 ± 4.89^c^	17.82 ± 1.34^a^	61.55 ± 0.79^e^	23.76 ± 0.98^d^
G3; 5% DSP	92.83 ± 0.92^d^	121.11 ± 10.06^c^	29.59 ± 2.47^b^	39.02 ± 2.71^d^	24.22 ± 2.01^d^
G4; 10% DSP	83.18 ± 2.53^c^	76.75 ± 2.63^b^	41.95 ± 1.96^c^	25.89 ± 2.26^c^	15.35 ± 0.53^c^
G5; 15% DSP	72.93 ± 1.31^b^	37.58 ± 2.63^a^	54.90 ± 2.50^d^	10.52 ± 3.45^b^	7.52 ± 0.53^b^

*Note:* Presented value levels are mean ± SD; *n* = 8 each group. Different superscript letters under the same column bearing are significantly different (*p* ≤ 0.05).

* Note that DSP is the powdered date seeds.

It can be noticed that the healthy control rats' group at G1 is the lowest in the total cholesterol levels, triglycerides, low‐density lipoprotein, and the very low‐density lipoprotein in all rat groups in contrast to the high‐density lipoprotein levels. Also, the most effective significant difference (*p* ≤ 0.05) in cholesterol levels was seen with group rats fed 15% DSP (G5; 72.93 ± 1.31 mg/dL). This CHO level is near to the negative control rats group fed a basal diet (G1; 68.37 ± 0.67 mg/dL). Again, the TG measured levels presented the lowest levels after 15% DSP consumption significantly (G5; 37.58 ± 2.63 mg/dL) versus the unhealthy positive control group (G2; 118.81 ± 4.89 mg/dL). HDL‐c is known as a good type of cholesterol (removes the extra cholesterol from the body; transporting it to the liver for excretion). This finally significantly lowers the risk of heart disease and stroke. Also, Table 4 illustrates the HDL‐c obtained in the experimental animals; the largest significant HDL‐c levels were seen within the rats group fed 15% DSP, followed by the rats fed 10% and then 5% DSP (54.90 ± 2.50, 41.95 ± 1.96, and 29.59 ± 2.47 mg/dL, respectively). On the other hand, the LDL‐c collected data (Table 4) were at the lowest levels with rats that consumed 15% DSP (10.52 ± 3.45 mg/dL). It is the closest significant group to the control healthy levels (4.16 ± 0.61 mg/dL). Moreover, the rats supplemented with 10% and 5% DSP presented different significant levels of LDL‐c according to the levels' consumption (5% > 10%; 39.02 ± 2.71 > 25.89 ± 2.26 mg/dL). Accordingly, and finally, the vLDL‐c levels depended on the acquired TG levels, as it has been measured by the following calculation (vLDL‐c = TG/5). The healthy control rats show 5.84 ± 0.15 mg/dL, which is about close to the last rat group (fed 15% DSP; 7.52 ± 0.53 mg/dL). Treated groups show 5% and 10% DSP supplementations significantly in very close levels (23.76 ± 0.98 and 24.22 ± 2.01 mg/dL, respectively), while the 15% DSP supplemented levels were the lowest significantly (15.35 ± 0.53 mg/dL; *p* ≤ 0.05). In conclusion, all measured lipid profiles levels in all collected data are in line with good correlated levels with DSP (Table 4). Lipid profile shows an improvement in diabetic animal models, especially rats that fed 15% DSP.

### 3.5 Kidney Functions

Collected serum samples have been used also for measuring the kidney functions (urea and creatinine) for all the experimental animal models and all obtained data have been recorded in the following Table 5. They have been measured to evaluate the renal injury; this could be correlated with high glucose levels causing renal function impairment or renal dysfunction. It can be noticed from Table 5 that kidney function referring to the measured urea and creatinine levels is at the highest levels among diabetic rat groups in comparison to the healthy control group (G1).

**TABLE 5:** Effect of DSP supplementation on kidney functions in all the experimental animal models.

Groups	Kidney functions (mg/dL) between used animal models				
	G1; −ve	G2; +ve	G3; 5% DSP	G4; 10% DSP	G5; 15% DSP
Urea	38.77 ± 1.16^a^	52.97 ± 1.56^d^	47.94 ± 1.52^c^	41.67 ± 1.53^b^	38.82 ± 0.69^a^
Creatinine	0.50 ± 0.09^a^	2.21 ± 0.10^d^	1.85 ± 0.12^c^	0.94 ± 0.04^b^	0.55 ± 0.03^a^

*Note:* Presented value levels are mean ± SD; *n* = 8 each group. Different superscript letters under the same row bearing are significantly different (*p* ≤ 0.05).

* Note that DSP is the powdered date seeds.

Regarding urea levels, it was shown that healthy control rats are 38.77 mg/dL versus 52.97 mg/dL for the diabetic control group (Table 5). The same table also illustrates the urea levels after different DSP supplementations (5%, 10%, and 15% DSP). It shows that the biggest effects come from 15% DSP additions significantly (*p* ≤ 0.05), that has been reduced to about similar levels for the control healthy group (38.82 ± 0.69 and 38.77 ± 1.16 mg/dL respectively). The lowest effective used doses on urea levels were in rat groups fed 5% significantly (*p* ≤ 0.05), then 10% of DSP (47.94 ± 1.52 and 41.67 ± 1.53 mg/dL respectively).

Moreover, Table 5 also illustrates the measured creatinine levels among animals in the current experiment. The positive healthy control rats were 0.50 ± 0.09 mg/dL significantly (*p* ≤ 0.05), that was the lowest compared to the diabetic rats (control positive group; 2.21 ± 0.10 mg/dL). However, the rats fed different DSP levels (5%, 10%, and 15%) showed 1.85 ± 0.12, 0.94 ± 0.04 and 0.55 ± 0.03 mg/dL respectively, significantly (*p* ≤ 0.05); the biggest effect at the high DSP supplemented levels (15%).

### 3.6 Gut Microbiota Composition

**FIGURE 1:**
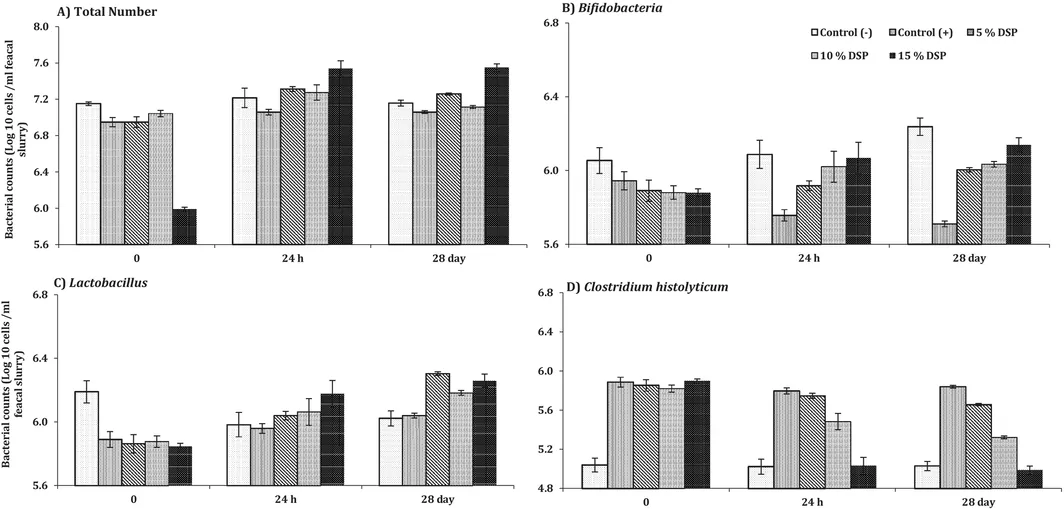
The colonic microbiota composition between used animal models at different time points (mean ± SD).

**FIGURE 2:**
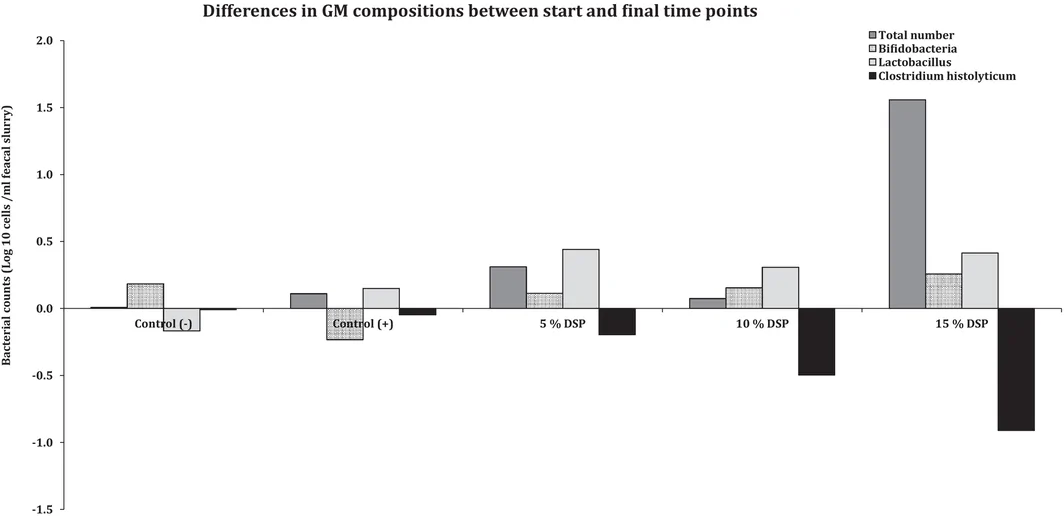
Differences of colonic bacterial levels between initial and final time points in all the animal models.

The measured gut microbiota in the current study was shown in the following Table 7 and following Figures 1 and 2. The measured microbiota of collected fecal samples are the total numbers of *Bifidobacteria, Lactobacillus*, and *Clostridium histolyticum* measured at different time points of the experiment (0 h, 24 h, and 28 days).

It can be noticed that the presented Table 6 and Figure 1 demonstrate that total microbiota numbers are the highest numbers in comparison to all measured species. The healthy control was nearly 7.17 Log 10 cells/mL fecal samples at the beginning of the experiment (0 h), while it was a little bit lower in the other diabetic groups (G2; +ve) at about 6.95 Log 10 cells/mL fecal samples at the same start time point (0 h; Table 6, Figure 1A). The diabetic animal rats were at high composition levels of total bacterial numbers within the group that fed 15% DSP, especially at the end of the experiment (after 28 days; significantly at *p* ≤ 0.05) at about 7.55 Log 10 cells/mL fecal slurry.

**TABLE 6:** Effects of date seeds powder (DSP) on gut microbiota compositions in used animal models.

Animal groups	Time points	Bacterial counts (Log 10 cells/mL feacal slurry)			
		Total number	*Bifidobacteria*	*Lactobacillus*	*Clostridium histolyticum*
G1; −ve	0	7.15 ± 0.02^b^	6.05 ± 0.07^b^	6.19 ± 0.23^b^	5.04 ± 0.04^a^
	24 h	7.21 ± 0.11^B^	6.09 ± 0.08^C^	5.98 ± 0.01^A^	5.02 ± 0.02^A^
	28 days	7.16 ± 0.03B	6.24 ± 0.05D	6.02 ± 0.24A	5.03 ± 0.02A
G2; +ve	0	6.95 ± 0.05^a^	5.94 ± 0.03^a^	5.89 ± 0.12^a^	5.89 ± 0.02^b^
	24 h	7.08 ± 0.03^A^	5.76 ± 0.14^A^	5.96 ± 0.07^A^	5.80 ± 0.08^C^
	28 days	7.06 ± 0.02A	5.71 ± 0.06A	6.04 ± 0.03A	5.80 ± 0.04D
G3; 5% DSP	0	6.95 ± 0.06^a^	5.89 ± 0.01^a^	5.86 ± 0.17^a^	5.85 ± 0.06^b^
	24 h	7.31 ± 0.03^B^	5.92 ± 0.02^B^	6.04 ± 0.03^A^	5.75 ± 0.04^C^
	28 days	7.26 ± 0.01C	6.00 ± 0.02B	6.20 ± 0.10B	5.66 ± 0.03C
G4; 10% DSP	0	7.04 ± 0.04^a^	5.88 ± 0.02^a^	5.88 ± 0.02^a^	5.82 ± 0.09^b^
	24 h	7.27 ± 0.08^B^	6.02 ± 0.07^bc^	6.06 ± 0.06^A^	5.48 ± 0.01^B^
	28 days	7.11 ± 0.02B	6.03 ± 0.05B	6.18 ± 0.04AB	5.32 ± 0.02B
G5; 15% DSP	0	6.99 ± 0.02^a^	5.88 ± 0.02^a^	5.85 ± 0.13^a^	5.90 ± 0.01^b^
	24 h	7.54 ± 0.09^C^	6.07 ± 0.04^bc^	6.18 ± 0.9^B^	5.03 ± 0.07^A^
	28 days	7.55 ± 0.04D	6.14 ± 0.03C	6.26 ± 0.13AB	4.99 ± 0.03A

*Note:* Presented value levels are mean ± SD; *n* = 8 each group. Different superscript small letters under the same column bearing are significantly different (*p* ≤ 0.05) at 0 h while CAPITAL letters are significantly differences in the same column for 24 h time point and also the CAPITAL Highlighted in the same column are significantly difference at 28 days of running the experimental.

* Note that PDS is the powdered date seeds.

Regarding the measured probiotic bacteria, especially the *Bifidobacteria* numbers, it can be noticed from Table 6 and Figure 1; B that *Bifidobacteria* numbers are at high levels in the healthy control group (G1; −ve). It counted about 6.20 Log 10 cells/mL fecal samples at 0 h, while it was at low levels in all diabetic rat groups (G2‐G5) at approximately 5.9 Log 10 cells/mL fecal samples. The diabetic rats in the control group for 24 h and 28 days (Table 6) and Figure 1 were at low levels (5.76 and 5.71 Log 10 cells/mL fecal samples respectively). DSP supplementation presented an increase in the *Bifidobacteria* numbers significantly (*p* ≤ 0.05), especially at the high supplemented DSP levels (15 > 10 > 5% DSP additions) over the long time (28 days; 6.14 > 6.03 > 6.00 Log 10 cells/mL fecal samples respectively), showing significant positive (*p* ≤ 0.05) prebiotic effects of the used DSP supplementation.

Additionally, *Lactobacillus* measured numbers have been recorded in Table 6 and presented in Figure 1. Again, it was in bigger levels in healthy diabetic rats' group (G1; 6.19 Log 10 cells/mL fecal samples) significantly (*p* ≤ 0.05) than the others diabetic rat groups at the start time point (0 h). The start times at diabetic control group (G2; +ve) show 5.89 Log 10 cells/mL fecal samples and also in diabetic other groups (G3, G4 and G5 at 5.86, 5.88, and 5.85 Log 10 cells/mL fecal samples respectively). However, the fed DSP restored numbers for *Lactobacillus* at all the used percentages significantly (*p* ≤ 0.05) of 5%, 10%, and 15% DSP additions (Table 6, Figure 1). It was increased at the end of the experiment (28 days) in all the diabetic groups (G3, G4 and G5; 6.30, 6.18 and 6.26 Log 10 cells/mL fecal samples, respectively).

On the other hand, *Clostridium histolyticum* was measured in all used animal models and it was illustrated again in Table 6 and presented in Figure 1. It was at low levels on the healthy control group (G1; −ve) in contrast to the other diabetic rats. *Lactobacillus* measured number was at 0 h of G1 at about 5 Log 10 cells/mL fecal samples while it was about 5.9 Log 10 cells/mL fecal samples with no significant changes on the all diabetic rats at the same 0 h point. The DSP additions show different decreased *Clostridium histolyticum* levels by the time within all supplemented concentrations (5%, 10%, and 15%) significantly (*p* ≤ 0.05). It was illustrated in Table 6 and Figure 1D and recorded levels at 28 days of running the experiment as follows: −G5 > −G4 > −G3; 4.09 < 5.32 < 5.66 Log 10 cells/mL fecal samples, respectively, significantly (*p* ≤ 0.05).

Moreover, Figure 2 demonstrates the differences significantly (*p* ≤ 0.05) in all measured gut microbiota compositions (GM; total numbers, *Bifidobacteria, Lactobacillus*, and *Clostridium histolyticum* ) between the start (0 h) and final time point (28 days) and concludes all effects of DSP supplemented levels. The most effects have been noticed within 15% DSP supplemented levels significantly (*p* ≤ 0.05), especially by *Clostridium histolyticum* reductions at 28 days. To conclude, the presented data in Table 6 and Figure 1A–D in addition to Figure 2 show that date seeds in powdered format have decreased the harmful colonic bacteria ( *Clostridium histolyticum* ) in contrast to improving some of the target probiotics species, mainly *Bifidobacteria* and *Lactobacillus*. This in total confirms that the intake of the supplemented DSP was associated with an increase of GM significantly (*p* ≤ 0.05).

### 3.7 Kidney Histology Structure

The collected kidneys were used for histological examination sections and have been demonstrated in Table 7. It has been presented in columns with group names, descriptions and pictures that in total focused on evaluating renal function and structure in all rat groups. G1 is the control healthy group (−ve). It has shown normal photomicrograph histological structure of kidney with normal renal parenchyma (H&E ×200) (Table 7A). Additionally, Table 7B shows the examined kidney of rats from the control diabetic group (+ve) showing atrophy of the glomerular tuft (black arrow), congestion of the renal blood vessel (blue arrow) and perivascular inflammatory cell infiltration (red arrow) (H&E ×400). Also, Table 7C–E shows that the kidneys of the rats group supplemented with 5%, 10%, and 15% DSP as C had slight congestion of glomerular tufts (black arrow) while D is a photomicrograph of a rat kidney that had no histopathological changes and E is a photomicrograph of a rat kidney that exhibited no histopathological lesions (H&E ×400).

**TABLE 7:** Histopathological of kidneys obtained from all the animal models where (A) is the examined samples from control healthy and (B) from control diabetic rats. (C–E) are kidneys from rats supplemented 5%, 10%, and 15% DSP respectively (H&E ×400). Note: DSP means date seeds powder.

Groups	Kidney histological examination (H&E ×200)	
	Meaning	Figures
G1; −ve	A: Photomicrograph of rat kidney showing the normal histological structure of renal parenchyma	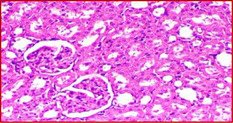
G2; +ve	B: Photomicrograph of rat kidney showing atrophy of glomerular tuft (black arrow), congestion of renal blood vessel (blue arrow) and perivascular inflammatory cells infiltration (red arrow)	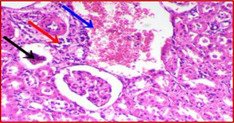
G3; 5% DSP	C: Photomicrograph of rat kidney showing slight congestion of glomerular tufts (black arrow)	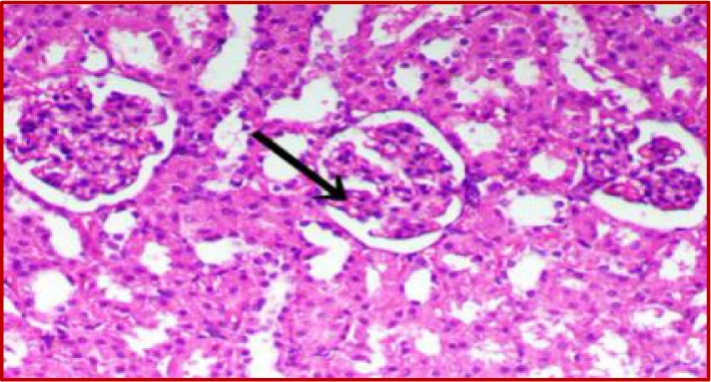
G4; 10% DSP	D: Photomicrograph of rat kidney showing no histopathological changes	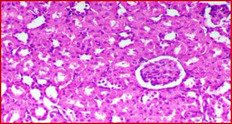
G5; 15% DSP	E: Photomicrograph of rat kidney exhibited no histopathological lesions	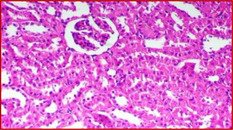

## 4 Discussion

Dates ( *Phoenix dactylifera* ) have many nutritional values in addition to huge antioxidant properties with especial high sugar content that could contribute to increasing global prevalence of diabetes (Butler et al. 2022). The date fruit is composed of seed and fleshy pericarp while seeds are between 13% and 15% of the date fruit weight. The worldwide levels of date production according to Food and Agriculture Organization Statistics (FAOSTAT; FAO 2023), have globally increased over the past decade (from 7.53 to 9.6 million metric tons) with about 852 thousand tons of seeds; waste by‐products (Jayasree et al. 2024). However, such seeds have shown different nutritional and health benefits of functional foods as a health promoting for either humans or animals (Al‐Thubiani and Khan 2017). Nowadays, and with increasing the novelty of prebiotic compounds, date seeds could be used to support the claim of using functional foods as an alternative approach for health promoting.

Date seeds in many different studies have been used for their potential health effect examinations in vivo and in vitro models because of their big levels of polyphenols and other bioactive compounds. The wasted date seed by product has high levels of different polyphenolic components like hesperidin, quercetin, kaempferol in addition to different phenolic acids especially allyl, epicatechin, catechol and chlorogenic with total dietary fiber especially pectin, β‐glucan, and arabinoxylan. It also has high levels of fats, proteins, minerals, and different other functional elements and nutrients (Moslemi et al. 2022). It has anti‐inflammatory activities that recommended it as a functional beverage for health status maintenance, immune systems improvement and chronic diseases prevention (Saryono et al. 2020; Moslemi et al. 2022). Indeed, the anti‐inflammatory effect of 2.5 g/day date seeds steeping consumed for 14 days in middle‐aged healthy women decreased the expression of different anti‐inflammatory mediators by down‐regulating pro‐inflammatory mediators' expression especially the IL‐1β, TGF‐β, COX‐1, and COX‐2 significantly (Saryono et al. 2020). However, no GM‐modulated studies have been conducted on diabetic models. Thus, the current study aims to measure the potential health effects of date seeds properties in association to GM compositions (valorization of date seed waste) in diabetic rats.

Firstly, the evaluated chemical composition of used date seed presented in Table 1 illustrates high dietary fiber levels of seed components. It also presents high sugars and different minerals such as potassium, iron, calcium, and sodium at high levels. Regarding the dietary fiber content, previous research illustrated that date seeds have dietary fiber to be considered for potential prebiotics health benefits (Al‐Thubiani and Khan 2017). Again, a study by Jayasree et al. (2024) demonstrated seeds' high levels of fibers (64–80 g/100 g). Also, recently published data shown that date seeds are rich in different elements, especially sugars, in addition to some bioactive compounds (carotenoids and flavonoids) (Alhomsi and Kılıç Bayraktar 2025). Such presented data of the chemical compositions also could cause the body weight recovery in diabetic rat groups (noted: DSP additions recovered the body weight comparing to the diabetic positive rat group).

About the evaluated blood biological analysis, glucose levels presented in Table 3, it was decreased after all the DSP supplementation with lowest effective DSP dose of rats group fed 5% DSP in contrast to the highest reduced glucose levels of rats fed 15% DSP. Such achievement with the blood glucose reduced levels shown in comparison as well to the diabetic control rats without any DSP treatment. Additionally, the lipid profile agrees with good correlated levels between each of used DSP. Lipid profile show an improvement between the used diabetic animal models especially rats fed 15% DSP for lowering the total cholesterol and triglycerides in addition to the HDL reduction in comparison to diabetic untreated animals. The good cholesterol knows as HDL is beneficial to the whole body. It helps for cholesterol collections returning it to the liver from tissues and the bloodstream then flushed from the body as waste (Abdessalem et al. 2024). The data presented herein agree with recent study by Jubayer et al. (2020) that found 600 mg of date seed powder supplementation for 90 days decreased total cholesterol, triglycerides, and low‐density lipoprotein in contrast to increasing the high‐density lipoprotein (Jubayer et al. 2020). Certainly, previous published studies showed that probiotics and/or prebiotics administration with different in vivo studies effectively improved the lipid profiles; especially total cholesterol, LDL and triglycerides reductions or of HDL increase (Ooi and Liong 2010; De Sales Guilarducci et al. 2020). Additionally, the measured kidney functions (urea and creatinine) used as biochemical markers for renal injury evaluation that could be affected by high glucose levels causing kidney damage and renal function impairment as indicators of renal dysfunction (Mohammed et al. 2020). Moreover, urea and creatinine levels after different DSP supplementations (5%, 10%, and 15%) show the biggest effects in urea levels with respect to 15% DSP additions. However, the creatinine biggest effective levels with rats were fed DSP supplemented at 15%. Such data are in correlation to collected data for blood glucose levels confirming the best high DSP dose for glucose and lipid profile presented herein and/or previously as discussed above.

Regarding the histological structure for collected kidney tissue, the histological structure for collected kidney tissue in all used rat groups presented a great correlation in agreement with measured lipid and blood glucose levels. The diabetic and low levels DSP presented signs of renal dysfunction as suggestive of diabetic‐related kidney damage while the 10% and 15% supplementations of DSP showed decreases in these pathological changes with no histological changes suggesting potential protective effects of DSP against diabetic models. Therefore, good correlation for lipid profile and kidney functions improvement as renal protective effects are in potential of DSP suggesting the anti‐diabetic effects of DSP consumption in order to improve the diabetic conditions and risk factors.

Finally, polysaccharides resist digestion till reaching the colon for nourishing beneficial gut bacteria or microbiota, especially the probiotics ones in order to their growth support with short‐chain fatty acid (SCFA) production (Mohamadizadeh et al. 2024; Han et al. 2022). The probiotics have been known for supporting the host's health when consumed in adequate amounts, such as lactic acid bacteria, especially *Lactobacilli* and *Bifidobacteria spp*., in different dairy products. The prebiotics beneficially affect the host in stimulating the growth and/or activity of GM selectively for different health promoting, thus host's health improvements. Both probiotics and prebiotics are termed as synbiotics when used in combination has acquired scientific recognition, though their applications as functional foods (Ooi and Liong 2010). The cheapest is the prebiotic dietary sources, which are being suggested for fermentative microbes and called fermentable soluble fibers. It can cause intestinal microflora modifications, promoting beneficial bacteria like *Bifidobacteria* and *Lactobacillus* to grow in contrast to *Bacteroides* and *Clostridium* decrease due to nutrition changes (Acevedo‐Román et al. 2024). This agrees with the data in this study for the measured GM that illustrates date seeds have deceased the harmful colonic bacteria ( *Clostridium histolyticum* ) in contrast to improving some of the probiotics species, mainly *Bifidobacteria* and *Lactobacillus*, suggesting an association of used treatment (DSP supplementations) and GM compositions. Such data are also in agreement with potentially carbon sources utilizations by the bacterial fermentation process that confirmed the potential use as a novel source of prebiotic by previous study for increasing probiotic *Lactobacillus* population (Al‐Thubiani and Khan 2017). Also, different data presented such great effects of DSP between diabetic models due to their dietary fibers. The dietary soluble fiber, such as β‐glucan (BG), shown hypoglycemic and hypo‐cholesterolemic effects with insulin resistance reduction, forming protective intestinal barrier and improving lipids and free cholesterol absorption (De Sales Guilarducci et al. 2020).

In conclusion, the current study recommends using date seeds as a prebiotics dietary source. They stimulate the growth of the probiotic as a carbon source extract for having potential health benefits. This is due to the presence of a large quantity of total dietary fibers affecting the colonic microbiota measured with lipid profile improvement and kidney functions in diabetic rats fed with DSP in different concentrations (5%, 10%, and 15%) with respect to 15% DSP supplementations.

## 5 Conclusion and Study Limitation

Date seed powder (DSP) aided diabetic rats and restored beneficial probiotic bacteria developments possessing prebiotic activities on the gut microbiota. This is due to the presence of total dietary fiber in large quantities for gut health or gut‐related disease management. This current study has revealed that DSP has beneficial effects; enhancing microbial growth in diabetic rats. The ideal dose is about 15% DSP supplementation estimated from the mean of all metabolic measured parameters. Therefore, DSP is a cheap source of prebiotics dietary fibers for functional food. It highlights the usage of sustainable waste by‐product into an economically viable food and carbon substrate source. However, different limitations are considered in the current study with especial reference to the FISH technique, numbers of measured GM and unmeasured SCFAs as GM metabolic end products.

## Author Contributions

**Huda Aljumayi:** validation, visualization, writing – review and editing. **Thamer Aljutaily:** software, formal analysis, validation, visualization, writing – review and editing. **Nazeha A. Khalil:** conceptualization, supervision, validation, writing – review and editing. **Belal M. Mohammed:** validation, visualization, writing – review and editing. **Asmahan A. Ali:** conceptualization, investigation, methodology, data curation, formal analysis, visualization. **Aeshah Alhosain:** validation, software, visualization, writing – review and editing. **Nouf Abdullah Alharbi:** validation, formal analysis, visualization, writing – review and editing. **Saleh A. Alsanie:** validation, resources, writing – review and editing, visualization. **Tarfa Albrahim:** investigation, methodology, visualization, resources, writing – review and editing, funding acquisition. **Isam A. Mohamed Ahmed:** validation, visualization, writing – review and editing.

## Funding

The work was supported by the Princess Nourah bint Abdulrahman University, Researchers Supporting Project number (PNURSP2026R69).

## Disclosure

*Declaration of Generative AI and AI‐Assisted Technologies in the Writing Process*: We confirm that no AI‐assisted technologies have been used in the current study.

## Ethics Statement

The current study has been approved ethically and biologically by the scientific research ethics committee (Animal Care and Use: 21‐SREC‐02‐2023). All authors declare that this research got conducted after being approved at the nutrition and food sciences department by the academic professional committee, Menoufia University, Egypt under safety and well‐being conditions.

## Conflicts of Interest

The authors declare no conflicts of interest.

## Supporting information


**Table S1:** The study groups of the experimental design.

## Data Availability

The data that support the findings of this study are available from the corresponding author upon reasonable request.
