# Correction to “Exploring the Functional Potential of Date (*Phoenix dactylifera*) Seed Bioactives in Modulating Gut Microbiota in Diabetic Rats”

**DOI:** 10.1002/fsn3.72407

**Published:** 2026-09-23

Alharbi, N. A. , S. A. Asanie , A. A. Ali , et al. 2026. Exploring the Functional Potential of Date (*Phoenix dactylifera*) Seed Bioactives in Modulating Gut Microbiota in Diabetic Rats. Food Science & Nutrition, 14, e71841. 10.1002/fsn3.71841.PMC1313659642088466

The name of the second author was misspelled and has been corrected from “Saleh A. Asanie” to “Saleh A. Alsanie”.

The online version of this article has been corrected accordingly.

We apologize for this error.

