# Supplementary material for: Exploring the Functional Potential of Date (Phoenix dactylifera) Seed Bioactives in Modulating Gut Microbiota in Diabetic Rats
Source: Food Sci Nutr. 2026 May 3;14(5):e71841. doi: 10.1002/fsn3.71841 (PMC13136596; doi:10.1002/fsn3.71841)
Supplement: Supplementary file 1 — Table S1: The study groups of the experimental design. [file FSN3-14-e71841-s001.docx]

**Table (A): The study groups of the experimental design**

| **Rat groups** | **Group descriptions** | **Group Code** |
| --- | --- | --- |
| **Control healthy** | Normal healthy rats; negative control group fed normal diet | **G1; *-ve*** |
| **Diabetic control** | Diabetic rats; positive control group fed normal diet | **G2; *+ ve*** |
| **Diabetic fed DSP** | Diabetic rats; positive control group fed normal diet with 5% DSP | **G3; 5% DSP** |
| **Diabetic fed DSP** | Diabetic rats; positive control group fed normal diet with 10% DSP | **G4; 10% DSP** |
| **Diabetic fed DSP** | Diabetic rats; positive control group fed normal diet with 15% DSP | **G5; 15% DSP** |

* Note that PDS is the powdered date seeds and n=8 each group (***P ≤ 0.05)***
